# 

**Published:** 1902-07

**Authors:** 


### PERSONALS.

Dr. W. C. Benner, of Doylestown, Pa., was a visitor to the Journal office in June.

The home of Dr. J. Payne Lowe, of Passaic, N. J., was recently blessed by the arrival of a son and prospective successor of his father.

Dr. S. L. Blount, of Temple, Texas, reports a much better state of affairs veterinary in his bailiwick. Fewer non-graduates than ever before. He reports the corn and cotton crop in great danger from drought.

Dr. James Mecray, of Maple Shade, N. J., met with the loss of his father in June. His father was a well-known practitioner for many years, and a graduate of the Medical Department of the University of Pennsylvania.

Dr. C. M. McFarland, of Billings, Montana, has entered the meat-inspection service of the Bureau of Animal Industry, and is now stationed at South St. Joseph, Missouri.

Dr. A. W. Ormiston, of Germantown, Philadelphia, is much interested in Free Masonry and a student of its mysteries and teachings through the school maintained at Philadelphia Masonic Temple.

Dr. F. Thatcher, located at El Paso, Texas, fills the rôle of Veterinary Inspector for the Mexican Government.

Dr. Geo. W. Kelm, of Milwaukee, Wis., is President of the Wolverine Mutual Live Stock Insurance Company, of Detroit, Michigan.

Dr. W. H. Yingst, formerly of Harrisburg, Pa., has received an appointment in the Bureau of Animal Industry, and is located at National Stock Yards, St. Clair Co., Illinois.

T. J. Ray, veterinarian, of Richfield, Minn., was chosen a lay juryman in one of Minneapolis' important trials involving the integrity of their city government.

Dr. R. G. Matthews, formerly of North Portal, has again located at Regina, Assa.

Dr. Elmer D. Nash, of Helena, Montana, is now Deputy State Veterinarian of that State, vice Dr. S. McClure, who has entered upon private practice.

Dr. J. S. Grant, of Portland, Mich., has removed to Mason City, Iowa, where he has erected a veterinary hospital and entered upon private practice.

Dr. Cecil French, of Washington, D. C., will spend the month of August at Bowery Beach on the coast of Maine.

Dr. McFarland, of Helena, Montana, a non-graduate practitioner, has been practising in that State more than forty years.

The Hollingsworth Veterinary Hospital of Utica, N. Y., is conducted by Dr. W. G. Hollingsworth as Medical Director, and Dr. W. A. Young as House Surgeon.

Dr. John J. Maher, of Philadelphia, Pa., gave a unique dinner of seventeen covers in the centre of his commodious veterinary hospital in June, in honor of the graduation of his student, Dr. John H. Zollinger, at the Veterinary Department of the University of Pennsylvania.

Dr. J. S. Pollard, of Providence, R. I., is Veterinarian to the State Board of Agriculture of the "Little Rhody" commonwealth.

Dr. J. M. Brodie, of Pontiac, Michigan, is a great admirer of the trotting bred horse.

Dr. William Dougherty, of Baltimore, Md., sailed from New York for Europe on the "Minnehaha" on May 10th. Dr. W. H. Rose, Prof. James L. Robertson, and Dr. Wm. Herbert Lowe were on the pier to see their old friend off and wish him bon voyage.

Dr. H. C. Shoemaker has formed a partnership with Dr. Joseph C. Fly, Philadelphia, Pa.

Dr. W. B. E. Miller, of Camden, N. J., will be associated with the extensive practice of Drs. Marshall and Fuller, of Philadelphia, in the future.

Dr. A. S. Shealy, who has been assistant to Dr. G. E. Nesom, of Clemson College, S. C., has obtained a leave of absence for one year and will return to the Veterinary Department of the Iowa State College to complete his course.

Dr. W. H. Dalrymple, of Baton Rouge, La., has just had conferred upon him an honorary membership in the Louisiana State Medical Association, in recognition of the distinguished services he has rendered in sanitary control work of animal diseases for the "Pelican State."

Dr. Geo. A. Waterman, Veterinarian of the Michigan Agricultural College, addressed the Michigan State Veterinary Medical Association on "The Relation of the Agricultural College to Veterinary Science.

Dr. F. J. Pagoda, an attache of the U. S. troop, is en route to Manila, P. I., from his former station at Fort Du Chesne, Utah.

Dr. H. S. Borneman, of Norristown, Pa., was a JOURNAL office visitor in June.

Dr. A. T. Sellers, of Camden, N. J., has been selected to fill the position of City Veterinarian. This place was recently created by an ordinance of councils.

